# Water deficit and potassium affect carbon isotope composition in cassava bulk leaf material and extracted carbohydrates

**DOI:** 10.3389/fpls.2023.1222558

**Published:** 2023-10-13

**Authors:** Jonas Van Laere, Roel Merckx, Rebecca Hood-Nowotny, Gerd Dercon

**Affiliations:** ^1^ Soil and Water Management & Crop Nutrition Laboratory, Joint FAO/IAEA Centre of Nuclear Techniques in Food and Agriculture, Department of Nuclear Sciences and Applications, International Atomic Energy Agency, Vienna, Austria; ^2^ Division of Soil and Water Management, Department of Earth and Environmental Sciences, KU Leuven, Heverlee, Belgium; ^3^ Institute of Soil Research, Department of Forest and Soil Sciences, University of Natural Resources and Life Sciences Vienna, Vienna, Austria

**Keywords:** carbon isotope discrimination, cellulose, drought, *Manihot esculenta* Crantz, nutrient stress, soluble sugar, starch, water use efficiency

## Abstract

Cassava (*Manihot esculenta* Crantz) is an important root crop, which despite its drought tolerance suffers considerable yield losses under water deficit. One strategy to increase crop yields under water deficit is improving the crop’s transpiration efficiency, which could be achieved by variety selection and potassium application. We assessed carbon isotope composition in bulk leaf material and extracted carbohydrates (soluble sugar, starch, and cellulose) of selected leaves one month after inducing water deficit to estimate transpiration efficiency and storage root biomass under varying conditions in a greenhouse experiment. A local and improved variety were grown in sand, supplied with nutrient solution with two potassium levels (1.44 vs. 0.04 mM K^+^) and were subjected to water deficit five months after planting. Potassium application and selection of the improved variety both increased transpiration efficiency of the roots with 58% and 85% respectively. Only in the improved variety were ^13^C ratios affected by potassium application (up to - 1.8‰ in δ^13^C of soluble sugar) and water deficit (up to + 0.6‰ in δ^13^C of starch and soluble sugar). These data revealed a shift in substrate away from transitory starch for cellulose synthesis in young leaves of the improved variety under potassium deficit. Bulk δ^13^C of leaves that had fully developed prior to water deficit were the best proxies for storage root biomass (r = - 0.62, r = - 0.70) and transpiration efficiency (r = - 0.68, r = - 0.58) for the local and improved variety respectively, making laborious extractions redundant. Results obtained from the youngest fully developed leaf, commonly used as a diagnostic leaf, were complicated by remobilized assimilates in the improved variety, making them less suitable for carbon isotope analysis. This study highlights the potential of carbon isotope composition to assess transpiration efficiency and yield, depending on the chosen sampling strategy as well as to unravel carbon allocation processes.

## 1 Introduction

In the light of climate change, cropping systems will require significant modifications to mitigate the negative impacts of altered and unpredictable rainfall patterns and rising temperatures. It will be imperative to adopt an integrated approach, combining the use of improved varieties with optimized agronomic practices. One crop which potentially has an important future role is cassava, as it maintains high production under stress conditions compared to other crops (Jarvis et al., 2012). Despite being well-known for its drought tolerance, storage root yield losses are nevertheless considerable when grown under conditions of water deficit (Connor et al., 1981).

A mechanism to optimize the utilization of limited water resources while increasing cassava’s resilience, could be by increasing its water-use efficiency (WUE) (Hatfield and Dold, 2019). WUE is a measure for how much water a crop uses per unit of biomass produced. It can be defined and measured at single leaf level (instantaneous WUE or intrinsic WUE), or at plant level (whole plant transpiration efficiency or economical transpiration efficiency) or at ecosystem level (Flexas et al., 2010). Increased WUE can be obtained by breeding better crop varieties and/or by changing management practices (Condon et al., 2004). Potassium application is such a management practice that has been suggested to increase WUE of cassava plants (Ezui et al., 2017). This is due to potassium’s generic role in stomatal functioning, osmotic adjustment, photosynthesis and other water regulating processes (Wang et al., 2013). Potassium application in cassava, a starchy root crop, is particularly important, since it mediates phloem loading, assimilate translocation (Oosterhuis et al., 2014) and starch synthesis (Cui and Tcherkez, 2021).

Leaf based approaches (stomatal conductance and CO_2_ assimilation) to assess WUE have been used for phenotyping, as they are mostly non-destructive, rapid and can be measured before harvest. They can considerably speed up breeding efforts (Condon et al., 2004). However, it is clear that care should be taken when extrapolating leaf-based estimations of WUE to whole plant WUE, because measurements at those two levels do not always agree (Seibt et al., 2008; Medrano et al., 2015). Moreover, a higher whole plant WUE does not always translate into more biomass under all given growth conditions (Blum, 2005).

Instantaneous measurements of WUE can only provide a snapshot of leaf gas-exchange and therefore, show considerable variation depending on microclimatic conditions (Lawson and Weyers, 1999). A more integrative measurement of leaf WUE is carbon isotope discrimination (Farquhar et al., 1982). Plants discriminate against the heavier isotope (^13^C) primarily through stomatal diffusion and carboxylation, leading to assimilates depleted in ^13^C compared to the atmosphere. Farquhar et al. established a (simplified) model that relates carbon isotope discrimination (CID, Δ^13^C) to the ratio of intercellular to atmospheric CO_2_ (c_i_/c_a_) as follows:


$${\text{Δ}}^{13}C=a+\left(b-a\right)*\frac{c_{i}}{c_{a}}$$

Where a stands for the fractionation due to differences in diffusivity of the isotopes through the stomata and b for the net fractionation due to carboxylation (Farquhar et al., 1989). Throughout the paper, δ^13^C will be used as an expression for carbon isotope composition, which is related to Δ^13^C as:


$${\text{Δ}}^{13}C=\;\frac{\delta^{13}C_{air}-\delta^{13}C_{sample}}{1+\delta^{13}C_{sample}}$$

With δ^13^C_air_ usually taken as -8‰ (Farquhar et al., 1989). A lower c_i_/c_a_, which can, for example, occur due to stomatal closure following drought, leads to less discrimination and therefore assimilates that are more enriched in ^13^C (higher or less negative δ^13^C, lower Δ^13^C). Like CID, intrinsic WUE depends on c_i_/c_a_, and therefore CID can be related to intrinsic WUE (Farquhar et al., 1982). Consequently, carbon isotope discrimination has been suggested as a phenotyping tool to select drought tolerant genotypes in multiple crops (Araus et al., 2015) and to assess the effects of agronomic practices like fertilizer application on WUE (Yi and Yano, 2022). The relationship between CID and whole plant WUE has been established in multiple crops, but with varying success as well as with opposite relationships (Medrano et al., 2015; Mininni et al., 2022). It is therefore important to test CID specifically for cassava, as no relationship between whole plant WUE and CID was established before.

Mostly, the carbon isotope signal in whole leaf (bulk) material is analyzed. However, within a leaf, carbon is present in different carbohydrate pools with different turn-over times. Hence, their δ^13^C signatures can be informative for different temporal scales (O’Leary, 1988). For instance, soluble sugars and starch (transitory starch) have been found to integrate plant physiological changes at a scale of one to two days (Brugnoli et al., 1988). Thereby starch is found usually enriched in ^13^C compared to soluble sugars because of the aldolase reaction which favors the heavier isotope (Gleixner et al., 1998). However, starch of storage organs, such as storage roots and stems of cassava, is likely to integrate the ^13^C signal during the period it was deposited, contrary to transitory starch which is stored during the day in chloroplasts and is broken down by the end of each night (Trethewey and Smith, 2000). Structural cellulose, which is assumed to be a non-reversible end product in plants (Hayashi et al., 2005), so has a δ^13^C signature determined by the mix of assimilates (freshly assimilated or remobilized) which are available during its formation (Kagawa and Battipaglia, 2022). Cellulose is predominantly deposited until the leaf is fully developed. Therefore, δ^13^C of cellulose would integrate c_i_/c_a_ over the time of leaf formation. However, this only holds true if assimilates for cellulose synthesis are derived from direct products of photosynthesis and not from remobilized older carbon reserves (Kagawa and Battipaglia, 2022).

Side by side comparisons of the effects of abiotic stressors on the carbon isotope composition of leaf structural as well as non-structural carbohydrate pools are scarce (Lauteri et al., 1993; Heintel et al., 2000), but might nevertheless provide valuable insights in plant physiological processes. Because extracting specific biochemical carbon compounds can be cumbersome and laborious, it is worthwhile to verify whether bulk isotope analysis could not give the same information on plant physiological responses.

Cassava exhibits specific behavior in terms of storage and remobilization of assimilates, as shown in (Duque and Setter, 2013; Van Laere et al., 2023), where carbohydrates stored in stems and petioles were slowly released to sustain growth of young tissues during abiotic stress events. This phenomenon could complicate the interpretation of carbon isotope data in youngest fully expanded leaves (4^th^ or 5^th^ leaf from the growing tip), which are often used as diagnostic leaves. Although several studies have used carbon isotope composition in cassava to assess abiotic stresses or water use efficiency (van der Merwe and Medina, 1991; El-Sharkawy and De Tafur, 2007; Burns et al., 2012; Vandegeer et al., 2012; Adjebeng-Danquah et al., 2016b; Brown et al., 2016; Maftukhah et al., 2023), these works are limited in number, and the approaches applied different in each. A range of plant organs has been used, including leaves from multiple canopy levels, roots and growing shoots, whilst evaluating effects of water deficit and variety selection. These studies have only focused on carbon isotopic composition of the bulk material, and none have explored the use of leaf extracted carbohydrates.

Therefore, the aim of this study is (1) to evaluate the utility and sampling techniques of stable carbon isotopes for predicting storage root biomass and transpiration efficiency (TE) including different carbohydrate pools (bulk, soluble sugar, starch and cellulose) of selected cassava leaves, as well as (2) to investigate the impact of variety and potassium application on the storage root biomass and transpiration efficiency of cassava plants under water deficit.

## 2 Material and methods

### 2.1 Plant materials and greenhouse conditions

Cutting clones from the varieties Narocass1 and Gacyacyari were obtained from the Rwanda Agriculture and Animal Resources Development Board and were grown in the greenhouses of the FAO/IAEA Soil and Water Management and Crop Nutrition Laboratory (Seibersdorf, Austria) between 6 November 2020 and 7 May 2021. Narocass1 is an improved variety and was released by NARO Uganda in 2015. It is tolerant to cassava mosaic disease and cassava brown streak disease (Manze et al., 2021) and has potential yields of 40-45 tons fresh roots per hectare (Abaca et al., 2021). Gacyacyari is a local, low yielding variety, which is commonly used in Rwanda (Night et al., 2011). Once in the greenhouse, 1 cm was removed from both ends of the cuttings to obtain a final uniform cutting size of 15 cm. Thereafter, one cutting was inserted vertically into each pot with a volume of 6 liters. The pots were filled with 9 kg of sand, which was washed with tap water (0.06-2 mm, bulk density of 1500 kg.m^-^³). Temperature and relative humidity in the greenhouse were controlled and logged (Onset HOBO MX1101) throughout the experiment and were 22.8 ± 4.9°C and 46.1 ± 12.8%, resulting in a Vapor Pressure Deficit of 1.63 ± 0.91 kPa. Greenhouse CO_2_ concentrations and δ^13^C-CO_2_ were monitored starting at 20 weeks after planting with Off-Axis Integrated-Cavity Output Spectroscopy (Off-Axis ICOS, Los Gatos Research, San Jose, CA, USA) in flow-through mode.

### 2.2 Experimental design

A randomized complete block design was used to compare the responses of both contrasting cassava varieties, i.e. Gacyacyari and Narocass1, to two watering regimes (90% vs 50% of pot capacity) and two potassium nutrient solutions (1.437 mM vs 0.036 mM K^+^), totaling eight treatment combinations. Each treatment combination was replicated on six tables (six blocks) in the greenhouse, giving a total of 48 plants.

At the start of the experiment, pot water holding capacity was determined for each separate pot by watering it to saturation and allowing free drainage until a constant weight was reached. This value was considered as the pot capacity. All plants were watered to weight with reverse osmosis water, to 90% of pot capacity three times a week. Starting at seventeen days after planting, 150 mL of treatment specific Steiner solution (Steiner (1961), see **Table S1** in the supporting information for formulation) was added with the water weight to achieve the correct pot weight three times a week until the end of the experiment. The high (K+) or low (K-) potassium treatments had a K^+^ concentration and potassium activity ratio (PAR) of 1.437 mM K^+^ and 1.475 mmol^0.5^.L^-0.5^, and 0.036 mM K^+^ and 0.028 mmol^0.5^.L^-0.5^, respectively, as outlined in **Table 1**. All pots were flushed with 2 liters of triple concentrated nutrient solution on 5 February and 10 March 2021, to avoid accumulation or deficiency of certain nutrients.

**Table 1:** Treatment combinations as used in this experiment and the corresponding watering and potassium treatments.

Treatment	Watering (from 5 MAP)	K concentration (mM)	Potassium Activity Ratio (mmol^0.5^.L^-0.5^)
**W+K+**	90% of pot capacity	1.437	1.475
**W+K-**	90% of pot capacity	0.036	0.028
**W-K+**	50% of pot capacity	1.437	1.475
**W-K-**	50% of pot capacity	0.036	0.028

K concentration and potassium activity ratio are given for the used nutrient solutions.

**Figure 1:**
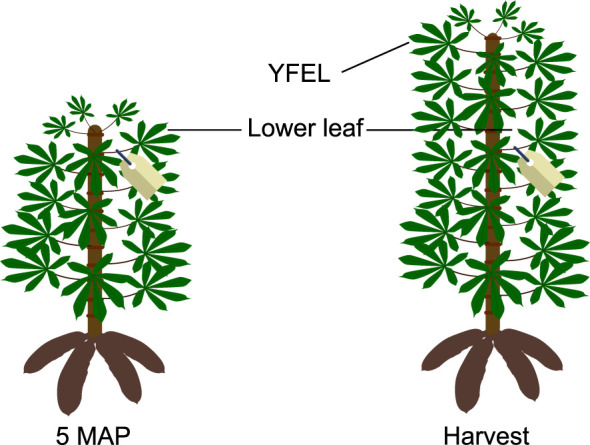
Selected leaves for stable carbon isotope analysis. At 5 months after planting (MAP), the youngest fully expanded leaves were tagged. At harvest, both the tagged leaf (named lower leaf in the text) and the youngest fully expanded leaf (YFEL) were sampled.

Five months after planting (MAP) water deficit was imposed on the plants of the W- treatment by applying water to 50% of the pot capacity, while W+ continued to receive water up to 90% of pot capacity. At this point, the youngest fully expanded leaf (YFEL) of each plant was tagged to identify which part of the shoot grew between 5 MAP and the end of the experiment at 6 MAP (see **Figure 1**). During the period of water deficit, irrigation frequency was increased to once per day to avoid water levels in pots of W+ plants to drop below 50%. Two pots without cuttings (one at 50% of pot capacity starting from 5 MAP and one at 90% of pot capacity throughout the experiment) were used per table to estimate evaporation to calculate plant transpiration.

### 2.3 Morphological and physiological parameters

Plant growth and functioning was assessed by both morphological and physiological parameters. Morphological parameters included plant height, number of nodes, number of leaves and stem diameter of each plant at the time of harvest. The percentage of leaf retention was obtained by dividing the number of leaves by the number of nodes and multiplying the result by 100. Physiological parameters included leaf temperature (FLIR E5-XT, Teledyne FLIR LLC, US), stomatal conductance (SC-1 leaf porometer, Meter group, Germany) and SPAD (SPAD-502, Konica Minolta, Japan) of the youngest fully expanded leaf between 11:00 and 15:00 at the start of drought, at mid drought (2 weeks of water deficit) and at the day before harvest (4 weeks of water deficit). A summary of these results can be found in the supporting information (**Tables S5**, **S6**).

### 2.4 Plant sampling procedure

At 6 MAP, plants were harvested block per block between 11:00 and 15:00, without receiving irrigation. At first, the first three youngest fully expanded leaf blades were sampled and immediately microwaved at 900W for 1 min to stop metabolic activity. The same procedure was used for the tagged leaves (youngest fully expanded leaves at 5 MAP, see (**Figure 1**). For the rest of the plant, leaf blades, petioles, stems, storage roots and fibrous roots were separated. Fresh weights of all parts were recorded, after which all samples were oven-dried at 55°C until constant weight to obtain dry weight. YFEL and tagged leaf (further referred to as lower leaf) samples were further ground to a fine powder with a micro ball mill (MM200, Retsch GmbH, Germany) for nutrient and isotope analyses. Harvest index was calculated as the ratio of storage root dry weight to total dry weight of the plants.

### 2.5 Plant water use and transpiration efficiency

Total transpiration (in L) of the plants was calculated based on the daily water gift subtracted by the daily evaporation from the empty pots. This was done for the period before drought (T_before drought_), during drought (T_drought_) and for the whole experimental period (T_total_). Whole plant transpiration efficiency (TE_whole plant_) and transpiration efficiency to create storage roots (TE_roots_) was calculated by dividing total biomass or root biomass of each plant by the transpiration of the whole experimental period respectively.

### 2.6 Leaf nutrient analysis

Fifty milligrams ground sample were acid digested in an open digestion block with 2 mL of 29% nitric acid solution in glass tubes for one hour at 90°C followed by seven hours at 120°C, while gently agitating the tubes. Digests were then diluted to 10 mL with MilliQ water. The resulting solution was analyzed using an inductively coupled plasma optical emission spectrometer (iCAP 7400 Duo, Thermo Scientific, China) at the Soil and Water Management Laboratories of KU Leuven, Belgium, to determine the macronutrient concentrations (P, K, Ca, S, Mg) in the leaves. A summary of nutrient concentrations can be found in the supporting information (**Tables S3**, **S4**).

### 2.7 Carbohydrate extractions

#### 2.7.1 Cellulose extraction

Aliquots of 200 ± 0.04 mg of the ground samples were transferred to PTFE bags (F57 filter bags, Ankom Technology, NY, USA) for further cellulose extraction using the modified Jayme-Wise method (Leavitt and Danzer, 1993). This method involves the removal of lignin by washing the samples repeatedly with an acidified sodium chlorite solution (10 g NaClO_2_ in 350 mL water acidified by 5 mL of 96% acetic acid), followed by multiple washes with 17% sodium hydroxide solution to remove hemi- and holocellulose and to obtain only α-cellulose. In this experiment, the washing procedure consisted of six 60-minute washes with the acidified chlorite solution, followed by three 45-minute washes with the sodium hydroxide solution and two additional 60-minute washes with the acidified chlorite solution all at 70°C in a shaking water bath, as modified from Andreu-Hayles et al. (2019).

#### 2.7.2 Extraction of soluble sugar

For the extraction of the neutral fraction of water-soluble compounds (referred to as soluble sugar hereafter), 51 ± 1 mg of each sample were transferred to 2 mL Eppendorf vials. The extraction was based on Wanek et al. (2001). Each sample was vortexed after receiving 1.5 mL of MilliQ water. All samples were placed in a shaking water bath (80°C) for a total of 30 minutes and were vortexed after 15 minutes. Vials were then centrifuged at 12000 g (Centrifuge 5415 C, Eppendorf) for two minutes. The pellet was used for starch extraction as described below. One milliliter of the supernatant was removed and pipetted on top of a column filled with anion (DOWEX 1X8, 100-200 mesh) and cation exchange (DOWEX 50WX8, 100-200 mesh) resin and eluted with 25 mL MilliQ water, to obtain the soluble sugar. These solutions were then freeze dried and resuspended in 1 mL of MilliQ water and stored at -20°C until further analysis.

#### 2.7.3 Starch extraction

The pellets, remaining after soluble sugar extraction, were stored at -20°C until starch extraction. Starch was extracted using enzymatic hydrolysis as described in Wanek et al. (2001). Pellets were thawed and resuspended in 1 mL of a methanol:chloroform:water (MCW) mixture (12:5:3 v:v:v) at 70°C for 30 minutes. Samples were then centrifuged for five minutes at 12000 g after which the supernatant was discarded. Two more washes with 1 mL MCW for ten minutes at 70°C were done. Samples were then put in the oven at 55°C overnight. Following this, 0.5 mL deionized water was added to the samples, and samples were kept in a water bath (100°C) for 30 minutes to gelatinize the starch. Thereafter, 0.5 mL of a solution with 1500 units.mL^-1^ of heat stable α-amylase was added to the samples, which were then kept at 85°C for 120 minutes in a water bath. Samples were again centrifuged for five minutes at 12000 g. Subsequently, 0.5 mL of the supernatant, containing the hydrolysate, was transferred to a new Eppendorf vial. To denature the α-amylase, 0.4 mL of chloroform was added to the sample and vortexed. Following ten minutes of centrifugation at 12000 g, the aqueous phase, containing dissolved glucose as the end-product of starch hydrolysis, was pipetted into tin capsules until it reached a weight between 0.2 and 0.3 mg. Samples were dried in the oven overnight at 55°C prior to isotope analysis.

### 2.8 Stable carbon isotope analyses

For carbon isotope analysis, 2.6 ± 0.3 mg (bulk), 0.5 ± 0.1 mg (soluble sugar), 0.22 ± 0.03 mg (starch) and 0.14 ± 0.04 mg (cellulose) of sample were weighed into tin capsules. δ^13^C values of these samples were determined with an elemental analyzer (Vario Isotope Select, Elementar, Germany) coupled to an isotope ratio mass spectrometer (isoprime 100, Elementar, Germany). All δ^13^C values were normalized on the Vienna-Pee Dee Belemnite scale by using two in-house calibrated standards (δ^13^C_VPDB_ = -26.07 ± 0.08‰; δ^13^C_VPDB_ = -10.95 ± 0.06‰), containing sucrose from sugar beet and sugarcane respectively. In-house standards were calibrated with IAEA-CH-6 (δ^13^C_VPDB_ = -10.449 ± 0.033‰) and IAEA-CH-7 (δ^13^C_VPDB_ = -32.151 ± 0.050‰). Repeated extraction and measuring of a quality control sample, consisting of cassava leaf material, resulted in δ^13^C_VPDB_ values of -24.62 ± 0.15‰ (n = 7, mean ± 1 standard deviation) for cellulose, -27.56 ± 0.02‰ (n = 10) for soluble sugar and -27.13 ± 0.10‰ (n = 10) for starch.

### 2.9 Data analysis

All treatment effects on yield attributes, water use, morphology and isotopic composition were assessed using linear mixed models to account for block effects with variety, water, potassium and all possible interactions as fixed effects, while table was used as a random effect (random intercept). For comparison of carbohydrate pools within a treatment group, we used ‘carbohydrate pool’ as a fixed effect and table and plant as a random effect. A type III analysis of variance was conducted using the Satterthwaite’s approximation to retrieve statistical significance of the fixed effects. All calculations were performed in R (version 4.1.3) (R Core Team, 2023) using the *lme4* package (Bates et al., 2015). Significance of pairwise differences between the carbohydrate pools were computed on the estimated marginal means with Tukey’s test by using the *emmeans* package. Pearson correlation coefficients and their corresponding significance between isotopic values and plant attributes were computed with the *stats* package in R.

The δ^13^C values which were most effective at predicting storage root biomass, transpiration efficiency and transpiration were identified with a percentile lasso approach (Roberts and Nowak, 2014) to account for the strongly correlated isotopic values and the limited sample size. This approach utilizes lasso regression (Tibshirani, 1996) as a predictor selection procedure, which uses L1 regularization, causing coefficients of less important predictors to shrink to zero. To establish stability with a relatively small sample size, we conducted cross-validation to determine the lambda value, a hyperparameter defining the degree of regularization, a hundred times and selected the lambda corresponding to the 75^th^ percentile. Computations were conducted in R using the cv.*glmnet* and *glmnet* functions of the *glmnet* package (Friedman et al., 2010). To compare importance of the selected predictors, the δ^13^C values were first centered and scaled with their standard deviations.

## 3 Results

### 3.1 Yield attributes

Total biomass, storage root biomass and number, and harvest index were affected by variety, potassium application and/or water deficit (see **Table 2**). Variety selection significantly (p< 0.001) affected all measured attributes except the total biomass and leaf biomass. The improved variety, Narocass1, showed an 87% increase in storage root production and a 0.19 increase in harvest index compared to the local variety, across all water and potassium treatments. However, the local variety, Gacyacyari, overall had a larger number of storage roots than Narocass1 with double the weight of fibrous roots.

**Table 2:** Yield attributes of two cassava varieties [Gacyacyari (local variety) and Narocass1 (improved variety)] under varying irrigation (90% or 50% of pot capacity) and potassium application (K+ and K- solution) at the end of the experimental period (6 MAP).

		Total Biomass	Storage roots	Harvest Index	Storage roots	Fibrous roots	Leaves
		(g dry weight)	(g dry weight)		(number plant^-1^)	(g dry weight)	(g dry weight)
Gacyacyari	W+K+	62.9 (15.7)	33.0 (17.4)	0.49 (0.20)	3.33 (1.86)	4.61 (1.69)	7.27 (0.59)
	W+K-	32.8 (4.9)	11.9 (5.4)	0.37 (0.16)	2.00 (1.10)	2.83 (0.67)	4.86 (1.38)
	W-K+	48.7 (8.4)	16.4 (10.4)	0.32 (0.15)	7.17 (1.83)	5.05 (0.97)	6.57 (0.56)
	W-K-	32.5 (7.3)	12.1 (6.9)	0.36 (0.16)	2.00 (1.41)	3.16 (1.10)	4.57 (0.95)
Narocass1	W+K+	75.0 (20.1)	52.0 (20.2)	0.67 (0.11)	3.00 (1.41)	2.70 (1.25)	7.88 (0.62)
	W+K-	19.4 (12.1)	14.5 (13.9)	0.37 (0.31)	1.33 (1.03)	1.15 (0.49)	4.25 (1.23)
	W-K+	74.7 (17.8)	54.7 (16.0)	0.73 (0.05)	3.00 (0.63)	2.37 (0.97)	6.44 (0.68)
	W-K-	28.5 (10.9)	15.9 (9.4)	0.50 (0.21)	2.00 (2.00)	1.55 (0.48)	5.47 (0.48)
variety (V)		ns	***	***	***	***	ns
water (W)		ns	ns	ns	**	ns	ns
potassium (K)		***	***	**	***	***	***
VxW		ns	ns	.	*	ns	ns
VxK		***	**	*	.	ns	ns
WxK		ns	ns	ns	ns	ns	**
VxWxK		ns	ns	ns	*	ns	*

Represented values are means (standard deviation). Number of observations is 5 for plants of Narocass1 under W+K- for total biomass and harvest index, and 6 for all other groups. Significance for each effect as a result from the type III ANOVA are given in the second part of the table.

p-values are given as.,*,**,*** corresponding with p< 0.1, 0.05, 0.01, 0.001 respectively. ns means non-significant.

Potassium application affected the performance of both varieties, leading to a higher total biomass. The biomass response was less for the local variety (+71%) compared to the improved variety (+207%), across the water regimes. Similar effects of potassium were found on storage root biomass (106% increase for Gacyacyari; 251% increase for Narocass1) and harvest index (0.04 increase for Gacyacyari; 0.26 increase for Narocass1), across the water regimes. Potassium application also significantly (p< 0.001) increased the number of storage roots and the fibrous root weight. Leaf biomass increased significantly (p< 0.001) for plants receiving more potassium. This increase in leaf biomass with potassium application was stronger for plants under optimal watering conditions (+66%) compared to plants under water deficit (+30%), across the water regimes.

A significant (p< 0.01) main effect of water deficit was only detected in the number of storage roots and this was mainly observed in the Gacyacyari variety. W-K+ plants of the local variety had four more storage roots under water deficit, compared to under non-water stressed conditions (W+K+).

### 3.2 Morphology

Observed treatment effects on plant height, number of nodes and leaf retention can be found in **Table 3**. Overall, the local variety (Gacyacyari) had a plant height that was 48% more compared to the improved variety (Narocass1), potassium application increased plant height by 39%, across varieties and watering regimes.

**Table 3:** Morphological parameters of two cassava varieties [Gacyacyari (local variety) and Narocass1 (improved variety)] under varying irrigation (90% or 50% of pot capacity) and potassium application (K+ and K- solution) at the end of the experimental period (6 MAP).

		Plant Height	Nodes	Leaf Retention
		(cm)	(number.plant^-1^)	(%)
Gacyacyari	W+K+	81 (16)	40 (3)	67 (12)
	W+K-	68 (16)	42 (5)	44 (6)
	W-K+	87 (14)	39 (4)	63 (8)
	W-K-	62 (13)	42 (2)	45 (3)
Narocass1	W+K+	66 (6)	38 (4)	69 (12)
	W+K-	37 (10)	40 (11)	62 (5)
	W-K+	57 (7)	41 (9)	54 (10)
	W-K-	41 (9)	34 (6)	57 (9)
variety (V)		***	ns	*
water (W)		ns	ns	*
potassium (K)		***	ns	***
VxW		ns	ns	.
VxK		ns	ns	***
WxK		ns	ns	ns
VxWxK		.	ns	ns

Represented values are means (standard deviation) of 6 observations. Significance levels for each effect as a result from the type III ANOVA are given in the second part of the table.

p-values are given as., * and *** corresponding with p< 0.1, 0.05 and 0.001 respectively. ns means non-significant.

Overall, Narocass1 had slightly more leaf retention (10% increase) compared to the local variety. Potassium application significantly (p< 0.001) increased the leaf retention by 21%, across varieties and watering regimes. The significant (p< 0.001) variety x potassium interaction effect demonstrates that the effect of potassium on leaf retention was more pronounced in the local variety (46% increase) compared to the improved variety (3% increase), across watering regimes. Leaf retention was also significantly (p< 0.05) affected by water deficit, leading to a 10% decrease in leaf retention in the W- plants, across varieties and potassium treatment. The number of nodes was not significantly affected by any of the treatments in this experiment.

### 3.3 Water use

In the period before the onset of water deficit, Gacyacyari (local variety) used overall significantly (p< 0.01) more water (17%) compared to Narocass1 (improved variety) (**Table 4**). No difference in transpiration between the two varieties was detected during the period starting after 5 MAP, when considering all water and potassium treatments. When the whole experimental period was considered, no difference between the two varieties was detected. Overall, plants that received more potassium had a significantly (p< 0.001) higher transpiration from planting until 5 MAP and from 5 until 6 MAP. This effect of potassium application on transpiration was stronger in Narocass1 (87% increase for K+) compared to Gacyacyari (34% increase for K+) in the first 5 MAP. A similar, but larger effect of potassium application was found on plant transpiration starting at 5 MAP, leading to a 216% increase in transpiration for Narocass1 when higher concentrations of potassium were applied, compared to only a 110% increase for Gacyacyari, when considering all water regimes.

**Table 4:** Water use attributes of two cassava varieties [Gacyacyari (local variety) and Narocass1 (improved variety)] under varying irrigation (90% or 50% of pot capacity) and potassium application (K+ and K- solution) at the end of the experimental period (6 MAP).

		T_total_	T_before drought_	T_drought_	TE_whole plant_	TE_roots_
		(L.plant^-1^)	(L.plant^-1^)	(L.plant^-1^)	(g biomass.L water^-1^)	(g biomass.L water^-1^)
Gacyacyari	W+K+	12.7 (1.4)	8.4 (1.4)	4.3 (0.5)	4.93 (0.94)	2.58 (1.27)
	W+K-	8.2 (1.4)	6.2 (1.0)	2.0 (0.7)	4.03 (0.30)	1.50 (0.71)
	W-K+	11.5 (1.3)	8.1 (1.1)	3.4 (0.3)	4.22 (0.35)	1.37 (0.75)
	W-K-	7.8 (1.8)	6.1 (1.1)	1.7 (0.8)	4.21 (0.62)	1.59 (0.88)
Narocass1	W+K+	13.1 (1.9)	8.2 (1.1)	4.9 (0.9)	5.66 (1.18)	3.89 (1.33)
	W+K-	5.2 (2.4)	3.9 (2.1)	1.2 (0.4)	3.86 (1.11)	2.34 (2.11)
	W-K+	12.1 (1.2)	7.9 (1.1)	4.2 (0.4)	6.10 (0.89)	4.46 (0.89)
	W-K-	6.3 (1.6)	4.7 (1.4)	1.6 (0.4)	4.38 (0.85)	2.35 (1.13)
variety (V)		.	**	ns	**	***
water (W)		ns	ns	*	ns	ns
potassium (K)		***	***	***	***	**
VxW		ns	ns	ns	ns	ns
VxK		**	*	**	*	.
WxK		ns	ns	*	ns	ns
VxWxK		ns	ns	ns	ns	ns

Represented values are means (standard deviation). Number of observations is 5 for plants of Narocass1 under W+K- for TE_whole plant_, and 6 for all other groups. Significance levels for each effect as a result from the type III ANOVA are given in the second part of the table.

p-values are given as.,*,**,*** corresponding with p< 0.1, 0.05, 0.01, 0.001 respectively. ns means non-significant.

Water deficit clearly affected transpiration during the drought period significantly (p< 0.05). Plants under water deficit transpired 12% less water overall than plants under optimal watering during the period of water deficit. The reduction in transpiration under water deficit was more pronounced for K+ plants, with a 17% decrease, while K- plants experienced even a small increase (5%) in transpiration or plant water loss under drought conditions, when considering both varieties.

Both whole plant transpiration efficiency (TE_whole plant_) and transpiration efficiency to create storage roots (TE_roots_) was significantly affected by variety selection (p< 0.01 and p< 0.001 respectively) and potassium application (p< 0.001 and p< 0.01 respectively) (**Table 4**). On average Narocass1 had a 16% higher TE_whole plant_ and an 85% higher TE_roots_ compared to Gacyacyari, across all water and potassium treatments. Potassium application caused a 58% increase in TE_roots_ and 27% increase in TE_whole plant_ across all treatments, and affected TE_whole plant_ more strongly in Narocass1 (42% increase) than in Gacyacyari (11% increase).

### 3.4 Carbon isotopic composition in bulk and extracted carbohydrates

The effects of water deficit and potassium application on the δ^13^C in the different carbohydrate pools and selected leaves, can be found in **Table 5**. As predicted, overall, water deficit significantly increased δ^13^C of the bulk leaf material (+ 0.3‰, p< 0.05), soluble sugar (+ 0.6‰, p< 0.01) and starch (+ 0.6‰, p< 0.05) in the lower leaf of Narocass1 (improved variety). In Gacyacyari (local variety), only marginally significant (p< 0.1) increases of δ^13^C were found for soluble sugar in the YFEL and cellulose in lower leaf for plants under water deficit.

**Table 5:** Leaf carbon isotope values (δ^13^C in ‰) as measured in bulk, soluble sugar, starch and cellulose extracted from YFEL and lower leaf for Gacyacyari (local variety) and Narocass1 (improved variety) at 6 MAP.

	Gacyacyari (local variety)							
	Youngest fully expanded leaf				Lower leaf			
	bulk	sugar	starch	cellulose	bulk	sugar	starch	cellulose
W+K+	-26.2±0.5^A^	-26.0±0.5^A^	-24.2±0.4^B^	-24.2±0.9^B^	-26.0±0.6^A^	-25.1±0.9^AB^	-23.8±0.7^C^	-24.2±0.7^BC^
W+K-	-26.1±0.4^A^	-25.4±1.0^A^	-24.1±0.8^B^	-24.2±0.3^B^	-25.8±0.3^A^	-24.7±0.5^B^	-23.9±0.5^C^	-23.8±0.3^C^
W-K+	-25.6±0.3^A^	-25.2±0.5^A^	-23.8±0.3^B^	-23.5±0.5^B^	-25.5±0.2^A^	-24.7±0.4^B^	-23.4±0.3^C^	-23.4±0.2^C^
W-K-	-26.1±0.7^A^	-25.0±0.8^B^	-24.5±0.8^BC^	-24.5±0.8^C^	-25.9±0.7^A^	-24.9±1.7^AB^	-24.1±1.0^B^	-24.0±0.7^B^
water (W)	ns	.	ns	ns	ns	ns	ns	.
potassium (K)	ns	ns	ns	.	ns	ns	ns	ns
W*K	ns	ns	.	.	.	ns	ns	**
	Narocass1 (improved variety)							
	Youngest fully expanded leaf				Lower leaf			
	bulk	sugar	starch	cellulose	bulk	sugar	starch	cellulose
W+K+	-26.3±0.6^A^	-25.4±0.8^B^	-24.1±0.5^C^	-24.1±0.7^C^	-26.3±0.2^A^	-25.4±0.4^B^	-24.0±0.3^C^	-24.3±0.4^C^
W+K-	-26.5±0.8^A^	-24.5±1.1^B^	-24.1±0.8^B^	-24.9±0.6^B^	-25.9±0.2^A^	-23.7±0.6^C^	-23.0±0.6^D^	-24.5±0.3^B^
W-K+	-25.9±0.3^A^	-25.0±0.3^B^	-23.9±0.2^C^	-23.7±0.6^C^	-26.2±0.4^A^	-24.9±0.8^B^	-23.6±0.6^C^	-24.4±0.8^BC^
W-K-	-26.3±0.6^A^	-24.2±0.4^C^	-24.1±0.7^C^	-24.7±0.6^B^	-25.4±0.3^A^	-23.0±0.4^C^	-22.1±0.9^D^	-24.3±0.5^B^
water (W)	ns	ns	ns	ns	*	**	*	ns
potassium (K)	ns	**	ns	**	***	***	***	ns
W*K	ns	ns	ns	ns	ns	ns	ns	ns

Represented values are mean ± standard deviation (Number of observations is 4 - 6 for cellulose and 6 for all other groups) per treatment. Significance levels for each effect as a result from the type III ANOVA are given in the lower part of the table. Different letters depict carbohydrate pools within a treatment group (only for horizontal comparison) that differ significantly (p< 0.05), based on Tukey’s test on estimated marginal means.

p-values are given as.,*,**,*** corresponding with p< 0.1, 0.05, 0.01, 0.001 respectively. ns means non-significant.

Significant, but opposite overall effects of potassium were found for soluble sugar and cellulose in the YFEL in Narocass1. While potassium application caused a decrease of δ^13^C in the soluble sugar (- 0.8‰, p< 0.01), it increased the δ^13^C in the cellulose (+ 0.9‰, p<0.01). A significant overall potassium effect was also found in the lower leaves. Here, a decrease was found in δ^13^C in the bulk (- 0.6‰, p< 0.001), soluble sugar (- 1.8‰, p< 0.001) and starch (- 1.2‰, p< 0.001) of the lower leaves of Narocass1 when applying potassium. No significant effects of potassium application were found in Gacyacyari. However, a significant (p< 0.01) interaction between water deficit and potassium application was found for the cellulose in the lower leaves in Gacyacyari, where water deficit increased the δ^13^C for plants that received more potassium and decreased/stayed equal for K- plants.

### 3.5 Relations between carbon isotopic composition and biomass & transpiration parameters

Correlations between δ^13^C of the bulk leaf material and extracted carbohydrates, and biomass and transpiration parameters are given in **Table 6**. Storage root biomass was negatively correlated with δ^13^C, where a significant (p< 0.05) correlation was found. The strongest correlation with storage root biomass in Gacyacyari (local variety) was found when compared with the bulk δ^13^C of the lower leaves (*r = -0.62 or 38% of the variance explained*). Whilst in the Narocass1 (improved variety) bulk δ^13^C (*r = -0.70 or 49% of the variance explained*) and soluble sugar fraction δ^13^C (*r = -0.72*) of the lower leaves gave the strongest correlations with storage root biomass. Total biomass was correlated with bulk δ^13^C of the lower leaf and with soluble sugar δ^13^C of both leaves for Narocass1, while it was just significantly correlated with the soluble sugar δ^13^C of both leaves only for Gacyacyari.

**Table 6:** Pearson correlation coefficients and their significance level for carbon isotopic composition (δ^13^C) of bulk, soluble sugar, starch and cellulose in youngest fully expanded leaves (YFEL) and lower leaf for Gacyacyari and Narocass1 with storage root biomass, total biomass, whole plant transpiration efficiency, transpiration efficiency to create storage roots and total transpiration.

	Gacyacyari (local variety)							
	Youngest fully expanded leaf				Lower leaf			
	bulk	sugar	starch	cellulose	bulk	sugar	starch	cellulose
Storage root biomass	-0.4°	-0.42*	-0.05	-0.4°	-0.62**	-0.45*	-0.31	-0.43*
Total biomass	-0.24	-0.5*	0.06	-0.15	-0.34	-0.41*	-0.18	-0.15
TE_whole plant_	-0.47*	-0.38°	-0.23	-0.47*	-0.68***	-0.44*	-0.35°	-0.52**
TE_roots_	-0.47*	-0.3	-0.15	-0.52*	-0.77***	-0.42*	-0.36°	-0.64***
T_total_	-0.03	-0.46*	0.22	0.15	-0.02	-0.27	0	0.11
	Narocass1 (improved variety)							
	Youngest fully expanded leaf				Lower leaf			
	bulk	sugar	starch	cellulose	bulk	sugar	starch	cellulose
Storage root biomass	-0.05	-0.48*	-0.18	0.25	-0.7***	-0.72***	-0.67***	-0.04
Total biomass	0	-0.49*	-0.16	0.35	-0.68***	-0.74***	-0.69***	0
TE_whole plant_	-0.13	-0.45*	-0.28	0.14	-0.58**	-0.58**	-0.53**	-0.09
TE_roots_	-0.31	-0.44*	-0.42*	-0.03	-0.59**	-0.51*	-0.52**	-0.24
T_total_	0.12	-0.42*	-0.03	0.43*	-0.63***	-0.71***	-0.67***	0.07

Number of observations is 22 for cellulose in the YFEL of Gacyacyari and the lower leaf in Narocass1, and 24 for all others.

Pearson correlation coefficients using pairwise complete observations with indicated significance levels (°,*,**,*** correspond to p< 0.1, 0.05, 0.01, 0.001 respectively).

Whole plant transpiration efficiency (TE_whole plant_) was significantly (p< 0.05) and negatively correlated with δ^13^C of bulk and multiple carbohydrate pools in both varieties. Strongest correlation for Gacyacyari were found in bulk δ^13^C of the lower leaf and for Narocass1 in the δ^13^C of bulk and soluble sugar in the lower leaf. We found strongest correlations with transpiration efficiency to create storage roots (TE_roots_) in bulk δ^13^C of the lower leaf of both varieties. Overall, no significant correlations were found between the bulk δ^13^C of the YFEL and any of the parameters in Narocass1.

The outcomes of the percentile LASSO regression can be found in **Table 7**. When combining all data, bulk δ^13^C of the lower leaves was selected as predictor for all variables, except for total transpiration. For Narocass1, bulk δ^13^C of the lower leaves was selected as a predictor for all variables, except for total transpiration. For transpiration and total biomass, δ^13^C in the soluble sugar of the lower leaves was indicated as most important predictor. For Gacyacyari, only TE_whole plant_ and TE_roots_ could be estimated through isotopic composition of bulk δ^13^C in the lower leaves when using LASSO regression.

**Table 7:** Selected predictors (δ^13^C) among carbohydrate pools (bulk, soluble sugar, starch and cellulose) by percentile LASSO regression in order of decreasing importance for the main variables of interest to predict total transpiration (T_total_), whole plant transpiration efficiency (TE_whole plant_), transpiration efficiency to create storage roots (TE_roots_) as well as total biomass and storage root biomass.

	Gacyacyari	Narocass1	Combined
T_total_	.	Sugar (lower) Starch (lower)	Sugar (lower) Cellulose (YFEL)
TE_roots_	Bulk (lower)	Bulk (lower)	Bulk (lower) Cellulose (lower)
TE_whole plant_	Bulk (lower)	Bulk (lower)	Bulk (lower)
Total biomass	.	Sugar (lower) Bulk (lower) Starch (lower)	Bulk (lower) Sugar (lower)
Storage root biomass	.	Bulk (lower) Sugar (lower) Starch (lower)	Bulk (lower)

‘.’ indicates instances where all variable effects where shrunk to 0.

**Figure 2:**
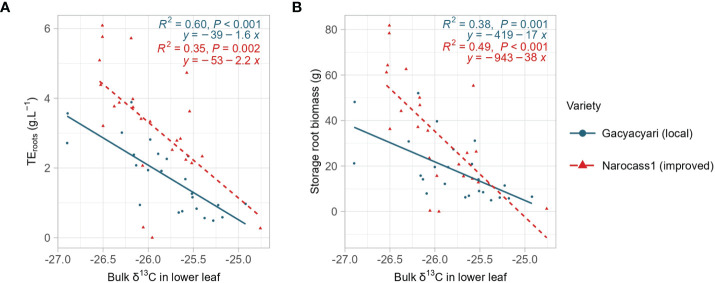
Analysis of covariance for bulk δ^13^C in the lower leaves and **(A)** transpiration efficiency to create storage roots (TE_roots_) or **(B)** storage root biomass. Blue color, solid line and dots represent Gacyacyari (local variety), red color and dashed line and triangles represent Narocass1 (improved variety).

Both storage root biomass and transpiration efficiency to create storage roots were significantly (p< 0.01) correlated with bulk δ^13^C of the lower leaves. Analysis of covariance showed (i) a significant (p< 0.05) difference in slope and intercept between the two varieties for the relationship between storage root biomass and bulk δ^13^C and (ii) a significant (p< 0.001) difference in intercept between both varieties for the relation between transpiration efficiency to create storage roots and bulk δ^13^C (**Figure 2**). Narocass1 had overall a higher transpiration efficiency to create storage roots linked to the same values of δ^13^C over the whole range of δ^13^C. At the same time, higher storage root biomass was found for the same δ^13^C in Narocass1 than in Gacyacyari at low levels of δ^13^C, but this effect declined at higher values of δ^13^C.

## 4 Discussion

Understanding how plants respond to water-limited and nutrient-poor conditions is critical for developing strategies to maintain crop productivity in the face of climate change. In this work, we tested stable carbon isotope composition in bulk leaf material and extracted carbohydrates of selected cassava leaves to assess physiological responses of cassava plants to water deficit and potassium availability.

### 4.1 Water deficit increases δ^13^C of direct photosynthetic products in the improved variety

Water deficit caused the predicted significant increases in δ^13^C (less negative) in the bulk, soluble sugar and starch fractions in the improved variety (Narocass1), but only in the lower leaves (**Table 5**). As cassava plants usually react to water deficit with rapid stomatal closure (El-Sharkawy, 2004), a reduction in c_i_/c_a_ was anticipated. According to the simplified model of Farquhar et al. (1989), this decreased c_i_/c_a_ results in increased δ^13^C (less negative), as observed in this experiment. Previous studies have similarly reported increased δ^13^C for cassava plants under drought. Roots sampled after one month of water deficit imposition (Brown et al., 2016) or third fully expanded leaves after two weeks of water deficit (Vandegeer et al., 2012), showed significant, but stronger increases of up to 1.5‰ and 1.9‰ respectively. Furthermore, Adjebeng-Danquah et al. (2016b) tested the effect of irrigation on δ^13^C for growing shoots of twenty varieties during the dry season. While the authors found no main effect of irrigation, a marginally significant interaction effect was found between irrigation and variety. This hints at irrigation affecting the δ^13^C of some varieties, but not of others, a finding similar in this experiment. The larger response of δ^13^C values to water deficit in soluble sugar and starch compared to bulk is also not surprising, since structural carbon fractions (such as cellulose) might mask the effect of water deficit, as these fractions were formed before the onset of water deficit.

### 4.2 Potassium application decreases δ^13^C of direct photosynthetic products in the improved variety

Potassium application decreased δ^13^C (more negative) of soluble sugar in the YFEL, as well as of bulk, soluble sugar and starch in the lower leaves of Narocass1 (improved variety) (**Table 5**). This decreased δ^13^C in soluble sugar and starch (direct products of photosynthesis) under increased potassium application, indicates an increased c_i_/c_a_ during assimilation of these products (Farquhar et al., 1989). Since an increase in photosynthesis of cassava is expected with increasing potassium availability (Omondi et al., 2020), a greater increase in stomatal conductance would be required to result in an increased c_i_/c_a_ (Farquhar et al., 1989). An increase in stomatal conductance with potassium application was indeed observed for YFEL of K+ plants of the Narocass1 variety (**Table S6** in supporting information). The effect of potassium on δ^13^C is a new finding for cassava, but similar to our findings, a decrease (0.28-2.7‰) in bulk δ^13^C for plants receiving more potassium was found before in cotton (Bednarz et al., 1998; Tsialtas et al., 2016) and highest δ^13^C values were found in plants with low K supply in spring wheat (Jákli et al., 2016). Nevertheless, opposite effects of K supply on δ^13^C potatoes (increases of <2.0‰) (Yi and Yano, 2022) as well as no effect on Hibiscus (Egilla et al., 2005) have also been found.

### 4.3 Potassium application may cause a shift in source assimilates for cellulose synthesis in the improved variety

Our results show that δ^13^C values of cellulose in Gacyacyari (local variety) leaves were almost identical to those found in starch, irrespective of the treatment (**Table 5**). In most cases soluble sugar had significantly lower δ^13^C values (more negative) than both starch and cellulose. We therefore hypothesize that the primary substrate for cellulose synthesis for Gacyacyari plants were assimilates obtained from starch degradation occurring at night. Previous research on Arabidopsis plants showing highest activities of enzymes related to cell wall synthesis during the final stages of the night, might further support this (Harmer et al., 2000).

The same pattern was seen for Narocass1 (improved variety) when receiving more potassium. However, under potassium deficiency, this variety showed a tendency to lower δ^13^C (more negative) values in leaf cellulose compared to the theoretically source soluble sugar and starch (Verbančič et al., 2018) in the same leaves. We hypothesize that potassium deficiency caused a shift in primary substrate for cellulose synthesis and caused K- plants of Narocass1 to rely more on carbohydrates remobilized from an unmeasured pool, already produced earlier, with lower δ^13^C values. Considerable import of old assimilates to youngest fully expanded leaves has been found before (Van Laere et al., 2023), but import quantity was independent of the imposed potassium treatments. However, it can be expected that less starch was available in the leaves of potassium deficient plants (Wasonga et al., 2020) and therefore possibly also less available for cellulose synthesis. The assimilates for cellulose synthesis might have been remobilized from stems and petioles, an unmeasured pool in our experiment, as they also serve as reserves under stress conditions (Duque and Setter, 2013).

These results demonstrate a significant potential for stable carbon isotope analysis of extracted carbohydrates to unravel source-sink relations in cassava. Cassava storage root yield is dependent on the production of assimilates as well as sink strength of the storage roots. Since both storage roots and growing tissues compete for available assimilates, varieties with stronger storage root sinks, might be beneficial. Further isotopic work, analyzing more possible carbon pools (e.g. roots, stems and petioles) as well as including labelling with ^13^C-CO_2_, could shed light on these source-sink relations and help crop improvement.

These new findings can be relevant for other crops as well, as they point out that interpreting cellulose isotope values needs to be done cautiously. Changes in cellulose δ^13^C can be caused by shifts in source substrate with a different δ^13^C, and therefore mask information on leaf WUE, as exemplified in the case for Narocass1.

### 4.4 Bulk δ^13^C of lower leaves as best proxy for yield and transpiration efficiency

The results of this experiment point towards the lower leaves as preferred diagnostic leaves when using stable carbon isotope composition. This follows from the simple correlations and LASSO regressions indicating bulk δ^13^C of the lower leaf as a good predictor for storage root biomass and transpiration efficiency (**Tables 6**, **7**). We found that up to 49% of the variance in storage root biomass was explained by bulk δ^13^C of the lower leaves. This finding is similar to the 49% (r = -0.70) explained variance as found in El-Sharkawy and De Tafur (2007) for bulk δ^13^C of 15 varieties averaged over three canopy levels. Contrary to our finding, no significant correlation between root yield and bulk δ^13^C of growing shoots was found in a study of Adjebeng-Danquah et al. (2016b). The positive relationship between δ^13^C and storage root biomass suggests a tight stomatal control as the main driver for differences in δ^13^C. Plants with higher δ^13^C, are those that have closed their stomata more often, having a lower CO_2_ supply for photosynthesis, resulting in lower yields (Condon et al., 2004).

In our experiment we found significant relationships between bulk δ^13^C of the lower leaf and whole plant transpiration efficiency as well as transpiration efficiency to create storage roots for both the improved and local variety (**Table 6**). The relationship between δ^13^C and transpiration efficiency has never been established before for cassava. The negative relationship found in this experiment is unexpected according to Farquhar et al. (1989). It suggests that intrinsic WUE (as estimated by δ^13^C) and whole plant transpiration efficiency are negatively correlated. This rather unexpected observation has been made before in grapevines (Medrano et al., 2015; Romero et al., 2023), alfalfa (Moghaddam et al., 2013; del Pozo et al., 2017) and prunus (Mininni et al., 2022), and it was suggested to be related to unproductive respiration and transpiration losses (Seibt et al., 2008; Konate et al., 2016).

Based on our results, we do not recommend the extraction of specific carbohydrate pools from lower leaves for estimating yield and transpiration efficiency, as there would be no additional benefit from the laborious extractions. However, lower leaves are not routinely used in cassava research and thus often unavailable, since youngest fully expanded leaves are preferred for diagnostic purposes such as nutrient analysis (Byju and Suja, 2020). Our experiment suggests that, in case only YFEL material is available, the analysis of δ^13^C in soluble sugar may still be more effective at revealing information on yield and whole plant transpiration efficiency (**Table 6**), but with the caveat they are also more time consuming and prone to error.

### 4.5 Stronger response to potassium for the improved variety

Our results show that, irrespective of the treatment, choosing the improved variety resulted in higher storage root biomass at 6 MAP. It was found that this was mainly the consequence of an increased allocation of assimilates to the roots for the improved variety, rather than a difference in total biomass production (**Table 2**). Furthermore, applying potassium was clearly more beneficial in the improved variety. This notion is supported by the stronger response of the improved variety to potassium application with larger increases in total biomass, storage root biomass, and harvest index in Narocass1 compared to the local variety.

Applying potassium generally increased whole plant transpiration efficiency of both the improved and local variety, while simultaneously increasing the total transpiration. These results suggest a proportionally larger increase in biomass production with potassium application. An observed higher leaf retention due to potassium application, which has been found before (Howeler, 1985), might have contributed to higher biomass production. The weaker response of Gacyacyari to potassium application for most of the parameters could also explain that no significant effects on carbon isotope composition could be found.

Even though no significant interaction was found between water deficit and potassium application for total biomass and storage root biomass (**Table 2**), a trend is visible in the local variety. However, this interaction effect is significant for the leaf dry weight (**Table 2**) and transpiration during the period of water deficit (**Table 4**). The effects of water deficit on total biomass and storage root biomass that can be observed for the local variety under the full potassium treatment - meaning lower dry weight with water deficit - cannot be observed with potassium deficiency. We hypothesize that the potassium deficit was so severe that it became more limiting than the water deficit.

### 4.6 Shortcomings and improvements

In this experiment, some factors might have hampered the estimation of whole plant transpiration efficiency based on isotope composition in the leaves of cassava plants. Firstly, we related isotope composition to the storage root biomass at 6 MAP. Even though correlations (R² = 0.29) have been previously found between storage root biomass at 6 MAP and 12 MAP (Adjebeng-Danquah et al., 2016a), it is plausible that the relation between carbon isotope composition at 6 MAP and storage root biomass at 12 MAP is rather tenuous. Cassava varieties are known to have different bulking behavior, which might complicate the interpretation of results at 6 MAP.

Secondly, the ratio of night-time transpiration to day-time transpiration remains unknown. Night-time transpiration rates have found to be as high as 30% of day-time transpiration rates (Snyder et al., 2003). As previously stated, unproductive transpiration could alter relationships between ^13^C (a proxy for intrinsic water use efficiency excluding night-time transpiration) and measured whole plant transpiration efficiency (which includes night-time transpiration). Estimations of this water loss could improve ^13^C-based estimations of whole plant transpiration efficiency (Konate et al., 2016).

Another issue is that the nutrient solutions were made according to Steiner (1961). To avoid different osmotic potentials in both solutions, we accounted for the loss of solutes in the K- nutrient solution by adding an amount of Ca^2+^ and Mg^2+^ so that K^+^ + Ca^2+^ + Mg^2+^ (3.335 mmol_c_.L^-1^) was the same for both solutions as well as the Ca^2+^: Mg^2+^ ratio (1.54:1). It is possible that the observed effects in this experiment are linked to variations in calcium and magnesium concentrations as well. Magnesium has been found to increase nitrogen uptake and plays a pivotal role in photosynthesis and phloem loading (Peng et al., 2020). A higher δ^13^C (less negative) of the direct photosynthetic products in the K- treatment, might therefore also be due to improved photosynthesis caused by increased Mg. However, opposite effects, meaning a lower δ^13^C with Mg application, have been found before (Tränkner et al., 2016). Calcium plays a role in cell wall stability and as a messenger (Thor, 2019). Direct effects of increased calcium on carbon isotope discrimination in the K- treatment are therefore less expected. Nonetheless, calcium and magnesium concentrations were considered sufficient in the K+ treatment, so a response to additional supply was not expected. Furthermore, it is important to note that the actual availability of potassium, as indicated by the potassium activity ratio, is even lower in the K- solution due to the addition of calcium and magnesium (1.475 mmol^0.5^.L^-0.5^ versus 0.028 mmol^0.5^.L^-0.5^).

Lastly, including a non-destructive approach with RGB color images to estimate fresh plant biomass, could further improve this experiment (Cabrera-Bosquet et al., 2016). This would allow water use efficiency to be estimated periodically throughout the experiment and account for growing biomass when irrigating plants gravimetrically.

## 5 Conclusion

The aim of this study was to evaluate the utility of carbon isotopes in various carbohydrate pools in two selected cassava leaves to assess transpiration efficiency and yield. We found that the youngest fully developed leaf, contrary to what is the usual in cassava research, was not the best diagnostic leaf. Translocation processes, providing youngest fully expanded leaves with remobilized assimilates, complicate interpretation and favor the older leaves for isotopic measurements. Nevertheless, it was shown that carbon isotope analysis of extracted carbohydrates in the youngest fully expanded leaves can be a useful tool to unravel carbon dynamics in cassava plants related to abiotic stresses. Further field experiments will have to confirm whether bulk δ^13^C of older leaves have the best predictive value for crop yield under field conditions.

The use of carbon isotope composition to predict transpiration efficiency and yield on crop level is promising, but interpreting δ^13^C data should be done with caution, as high leaf-based transpiration efficiency (δ^13^C) translated into low whole plant transpiration efficiency.

Both potassium application and variety selection could play a significant role in increasing cassava’s transpiration efficiency. However, a strategy where both improved varieties and potassium application are used, seems to be the most beneficial.

## Data availability statement

The original contributions presented in the study are publicly available. This data can be found here: https://doi.org/10.5281/zenodo.7929990.

## Author contributions

JL: conceptualization, methodology, investigation, data curation, formal analysis, writing – original draft. RM: conceptualization, methodology, writing – review & editing. RH-N: conceptualization, methodology, writing – review & editing. GD: conceptualization, methodology, writing – review & editing, project administration, supervision. All authors contributed to the article and approved the submitted version.
