# Supplementary material for: Water deficit and potassium affect carbon isotope composition in cassava bulk leaf material and extracted carbohydrates
Source: Front Plant Sci. 2023 Oct 13;14:1222558. doi: 10.3389/fpls.2023.1222558 (PMC10611503; doi:10.3389/fpls.2023.1222558)
Supplement: Supplementary file 1 [file Table_1.docx]

Supplementary Material

Water deficit and potassium affect carbon isotope composition in cassava bulk leaf material and extracted carbohydrates

Jonas Van Laere*, Roel Merckx, Rebecca Hood-Nowotny, Gerd Dercon

*** Correspondence:** Jonas Van Laere: jonas.vanlaere@kuleuven.be

# Steiner Solution

Table S1 Molarities of the different compounds in the adjusted Steiner solutions of the two potassium treatments (K+ and K- solution). Stock solutions with different compounds were first made mixed as according to Steiner before each watering event.

| Compound | K+ solution | K- solution | Unit |
| --- | --- | --- | --- |
| Ca(NO_3_)_2_.4H_2_O | 0.576 | 0.914 | mM |
| KNO_3_ | 1.226 | 0 | mM |
| MgSO_4_.7H_2_O | 0.373 | 0.373 | mM |
| KH_2_PO_4_ | 0.200 | 0.026 | mM |
| Ca(H_2_PO_4_)_2_.H_2_O | 0 | 0.087 | mM |
| Mg(NO_3_)_2_.6H_2_O | 0 | 0.275 | mM |
| H_3_BO_3_ | 8.701 | 8.701 | µM |
| MnSO_4_.H_2_O | 2.367 | 2.367 | µM |
| ZnSO_4_.7H_2_O | 0.352 | 0.352 | µM |
| Na_2_MoO_4_.2H_2_O | 0.104 | 0.104 | µM |
| CuSO_4_.5H_2_O | 0.062 | 0.062 | µM |
| C_10_H_14_O_8_N_2_Na_2_.2H_2_O (EDTA) | 8.951 | 8.951 | µM |
| KOH | 10.373 | 10.373 | µM |
| FeSO_4_.7H_2_O | 8.949 | 8.949 | µM |
| H_2_SO_4_ | 0.200 | 0.200 | µM |

Table S2 Total ion concentration (in mM) of the macronutrients in the adjusted Steiner solutions of the two potassium treatments (K+ and K- solution). Micronutrient concentrations were identical in both solutions.

| Ion | K+ solution | K- solution | Unit |
| --- | --- | --- | --- |
| NO_3_^-^ | 2,378 | 2,378 | mM |
| K^+^ | 1,436 | 0,036 | mM |
| Ca^2+^ | 0,576 | 1,001 | mM |
| SO_4_^2-^ | 0,385 | 0,385 | mM |
| Mg^2+^ | 0,373 | 0,648 | mM |
| HPO_4_^2-^ | 0,200 | 0,200 | mM |

# Leaf nutrient contents

Table S3 Leaf nutrient concentrations of the youngest fully expanded leaves at harvest (6 MAP) of two cassava varieties (Gacyacyari (local variety) and Narocass1 (improved variety)) under varying irrigation (90% or 50% of pot capacity) and potassium application (K+ and K- solution). Represented values are means (standard deviation). Number of observations is 6. Significance levels for each effect as a result from the type III ANOVA are given in the second part of the table.

|  |  | N | P | K | S | Mg | Ca |
| --- | --- | --- | --- | --- | --- | --- | --- |
|  |  | % | % | % | % | % | % |
| Gacyacyari | W+K+ | 2,77 (0,14) | 0,21 (0,02) | 1,17 (0,14) | 0,18 (0,01) | 0,39 (0,04) | 0,98 (0,19) |
|  | W+K- | 3,20 (0,24) | 0,28 (0,03) | 0,40 (0,08) | 0,19 (0,02) | 0,82 (0,16) | 1,93 (0,79) |
|  | W-K+ | 2,94 (0,37) | 0,21 (0,01) | 1,31 (0,21) | 0,20 (0,02) | 0,41 (0,07) | 0,85 (0,22) |
|  | W-K- | 3,31 (0,14) | 0,28 (0,03) | 0,39 (0,05) | 0,21 (0,02) | 0,81 (0,26) | 1,67 (0,73) |
| Narocass1 | W+K+ | 3,01 (0,33) | 0,24 (0,02) | 1,15 (0,11) | 0,20 (0,02) | 0,41 (0,06) | 1,20 (0,19) |
|  | W+K- | 3,41 (0,25) | 0,35 (0,05) | 0,45 (0,08) | 0,25 (0,02) | 0,93 (0,15) | 2,44 (0,48) |
|  | W-K+ | 3,34 (0,27) | 0,25 (0,02) | 1,06 (0,04) | 0,23 (0,02) | 0,45 (0,10) | 1,26 (0,23) |
|  | W-K- | 3,29 (0,18) | 0,31 (0,03) | 0,41 (0,08) | 0,21 (0,03) | 1,04 (0,26) | 2,26 (0,38) |
| variety (V) | | ** | *** | ns | *** | * | ** |
| water (W) | | . | ns | ns | . | ns | ns |
| potassium (K) | | *** | *** | *** | * | *** | *** |
| VxW | | ns | ns | * | ns | ns | ns |
| VxK | | ns | ns | ** | ns | ns | ns |
| WxK | | . | ns | ns | ** | ns | ns |
| VxWxK | | ns | ns | ns | ** | ns | ns |

p-values are given as . ,*,**,*** corresponding with p < 0.1, 0.05, 0.01, 0.001 respectively. ns means non-significant.

Table S4 Leaf nutrient concentrations of the lower leaves at harvest (6 MAP) of two cassava varieties (Gacyacyari (local variety) and Narocass1 (improved variety)) under varying irrigation (90% or 50% of pot capacity) and potassium application (K+ and K- solution). Represented values are means (standard deviation). Number of observations is 6. Significance levels for each effect as a result from the type III ANOVA are given in the second part of the table.

|  |  | N | P | K | S | Mg | Ca |
| --- | --- | --- | --- | --- | --- | --- | --- |
|  |  | % | % | % | % | % | % |
| Gacyacyari | W+K+ | 2,51 (0,23) | 0,23 (0,05) | 1,23 (0,17) | 0,17 (0,02) | 0,73 (0,22) | 1,84 (0,79) |
|  | W+K- | 2,54 (0,38) | 0,29 (0,13) | 0,23 (0,12) | 0,14 (0,06) | 1,04 (0,47) | 1,95 (0,91) |
|  | W-K+ | 2,55 (0,28) | 0,21 (0,02) | 1,39 (0,36) | 0,17 (0,02) | 0,69 (0,09) | 1,62 (0,35) |
|  | W-K- | 2,39 (0,56) | 0,32 (0,09) | 0,20 (0,09) | 0,17 (0,02) | 1,23 (0,13) | 2,14 (0,35) |
| Narocass1 | W+K+ | 2,77 (0,18) | 0,24 (0,02) | 1,10 (0,13) | 0,18 (0,02) | 0,74 (0,11) | 2,16 (0,35) |
|  | W+K- | 2,83 (0,17) | 0,39 (0,10) | 0,38 (0,04) | 0,25 (0,06) | 1,45 (0,13) | 3,66 (0,41) |
|  | W-K+ | 2,92 (0,56) | 0,26 (0,04) | 1,10 (0,31) | 0,20 (0,03) | 0,77 (0,32) | 2,25 (0,77) |
|  | W-K- | 2,87 (0,19) | 0,39 (0,05) | 0,37 (0,04) | 0,20 (0,01) | 1,51 (0,20) | 3,16 (0,34) |
| variety (V) | | *** | ** | ns | *** | ** | *** |
| water (W) | | ns | ns | ns | ns | ns | ns |
| potassium (K) | | ns | *** | *** | ns | *** | *** |
| VxW | | ns | ns | ns | ns | ns | ns |
| VxK | | ns | ns | ** | * | * | * |
| WxK | | ns | ns | ns | ns | ns | ns |
| VxWxK | | ns | ns | ns | * | ns | ns |

p-values are given as . ,*,**,*** corresponding with p < 0.1, 0.05, 0.01, 0.001 respectively. ns means non-significant.

# Physiological Measurements

Table S5 Stomatal conductance (g_s_), leaf temperature (T_leaf_) and SPAD of the mid lobe of the youngest fully expanded leaf of Gacyacyari (local variety) at the beginning of drought (5 MAP), mid drought (two weeks of water deficit) one day before harvest (before and after irrigation). All values were measured between 11:00 and 15:00. Represented values are means (standard deviation). Number of observations is 6. Significance levels for each effect as a result from the type III ANOVA are given in the second part of the table.

|  |  | Begin drought | | | Mid drought | | | Harvest (before irrigation) | | | Harvest (after irrigation) | |
| --- | --- | --- | --- | --- | --- | --- | --- | --- | --- | --- | --- | --- |
|  |  | g_s_ | T_leaf_ | SPAD | g_s_ | T_leaf_ | SPAD | g_s_ | T_leaf_ | SPAD | g_s_ | T_leaf_ |
|  |  | (mmol H_2_O.cm^-2^.s^-1^) | (°C) |  | (mmol H_2_O.cm^-2^.s^-1^) | (°C) |  | (mmol H_2_O.cm^-2^.s^-1^) | (°C) |  | (mmol H_2_O.cm^-2^.s^-1^) | (°C) |
| Gacyacyari | W+K+ | 156 (14) | 19.2 (0.3) | 33.7 (1.1) | 292 (24) | 21.3 (0.3) | 34.3 (0.5) | 417 (47) | 27.9 (0.3) | 32.6 (0.6) | 484 (37) | 21.3 (0.2) |
|  | W+K- | 49 (6) | 20.5 (0.2) | 32.5 (0.4) | 248 (35) | 22.0 (0.2) | 31.2 (0.7) | 319 (31) | 28.3 (0.3) | 31.5 (0.7) | 346 (23) | 21.7 (0.2) |
|  | W-K+ | 100 (9) | 21.2 (0.3) | 35.9 (0.5) | 200 (9) | 21.7 (0.2) | 35.7 (0.6) | 255 (25) | 29.2 (0.4) | 32.5 (1.1) | 335 (21) | 21.6 (0.3) |
|  | W-K- | 65 (6) | 20.5 (0.3) | 34.0 (0.6) | 187 (18) | 22.6 (0.2) | 29.1 (0.5) | 406 (66) | 27.5 (0.3) | 31.1 (0.5) | 415 (60) | 21.8 (0.3) |
| water (W) | | ns | ns | ns | ns | ns | ns | ns | ns | ns | ns | ns |
| potassium (K) | | ** | ns | ns | ns | . | * | ns | ns | ns | ns | ns |
| WxK | | ns | ns | ns | ns | ns | ns | ns | * | ns | ns | ns |

p-values are given as . ,*,**,*** corresponding with p < 0.1, 0.05, 0.01, 0.001 respectively. ns means non-significant.

Table S6 Stomatal conductance (g_s_), leaf temperature (T_leaf_) and SPAD of the mid lobe of the youngest fully expanded leaf of Narocass1 (improved variety) at the beginning of drought (5 MAP), mid drought (two weeks of water deficit) one day before harvest (before and after irrigation). All values were measured between 11:00 and 15:00. Represented values are means (standard deviation). Number of observations is 6. Significance levels for each effect as a result from the type III ANOVA are given in the second part of the table

|  |  | Begin drought | | | Mid drought | | | Harvest (before irrigation) | | | Harvest (after irrigation) | |
| --- | --- | --- | --- | --- | --- | --- | --- | --- | --- | --- | --- | --- |
|  |  | g_s_ | T_leaf_ | SPAD | g_s_ | T_leaf_ | SPAD | g_s_ | T_leaf_ | SPAD | g_s_ | T_leaf_ |
|  |  | (mmol H_2_O.cm^-2^.s^-1^) | (°C) |  | (mmol H_2_O.cm^-2^.s^-1^) | (°C) |  | (mmol H_2_O.cm^-2^.s^-1^) | (°C) |  | (mmol H_2_O.cm^-2^.s^-1^) | (°C) |
| Narocass1 | W+K+ | 179 (14) | 20.5 (0.3) | 35.9 (0.6) | 465 (29) | 20.8 (0.1) | 36.9 (0.5) | 499 (24) | 27.0 (0.3) | 32.7 (0.4) | 480 (21) | 21.2 (0.3) |
|  | W+K- | 92 (9) | 20.8 (0.3) | 33.5 (0.6) | 287 (29) | 21.4 (0.2) | 32.6 (0.7) | 276 (30) | 27.2 (0.3) | 30.7 (0.4) | 309 (32) | 21.8 (0.2) |
|  | W-K+ | 218 (17) | 19.3 (0.1) | 37.0 (0.3) | 455 (30) | 21.2 (0.1) | 36.3 (0.6) | 244 (22) | 27.8 (0.4) | 34.1 (0.5) | 494 (26) | 21.3 (0.1) |
|  | W-K- | 118 (11) | 19.9 (0.2) | 37.0 (0.5) | 250 (14) | 21.9 (0.2) | 33.2 (0.4) | 196 (12) | 28.7 (0.3) | 28.2 (0.3) | 274 (19) | 22.4 (0.2) |
| water (W) | | ns | . | . | ns | * | ns | ** | * | ns | ns | ns |
| potassium (K) | | ** | ns | ns | ** | * | * | * | ns | *** | ** | . |
| WxK | | ns | ns | ns | ns | ns | ns | ns | ns | . | ns | ns |

p-values are given as . ,*,**,*** corresponding with p < 0.1, 0.05, 0.01, 0.001 respectively. ns means non-significant.
